# The effects of marine heatwaves on a coral reef snapper: insights into aerobic and anaerobic physiology and recovery

**DOI:** 10.1093/conphys/coae060

**Published:** 2024-08-21

**Authors:** Shannon J McMahon, Philip L Munday, Jennifer M Donelson

**Affiliations:** ARC Centre of Excellence for Coral Reef Studies, James Cook University, 1 James Cook Dr, Douglas, Townsville, Queensland, Australia, 4814; Marine Climate Change Unit, Okinawa Institute of Science and Technology, 1919-1 Tancha, Onna, Okinawa, Japan, 904-0412; ARC Centre of Excellence for Coral Reef Studies, James Cook University, 1 James Cook Dr, Douglas, Townsville, Queensland, Australia, 4814; ARC Centre of Excellence for Coral Reef Studies, James Cook University, 1 James Cook Dr, Douglas, Townsville, Queensland, Australia, 4814

**Keywords:** Aerobic metabolism, lactate, recovery, haemoglobin, capture stress

## Abstract

Marine heatwaves (MHWs) are increasing in frequency and intensity. Coral reefs are particularly susceptible to MHWs, which cause mass coral bleaching and mortality. However, little is known about how MHWs affect coral reef fishes. Here, we investigated how MHWs affect the physiology of a coral reef mesopredator, *Lutjanus carponotatus*. Specifically, we exposed mature adults to two different MHW intensities, +1°C (29.5°C) and + 2°C (30.5°C) and measured physiological performance at 2 and 4 weeks of exposure and at 2 weeks post-exposure. At these time points, we measured oxygen consumption at rest and after a simulated fishing capture event, recovery time, excess post-exercise oxygen consumption (EPOC) and associated biochemical markers in the blood (baseline lactate, post-capture lactate, glucose, haemoglobin levels and haematocrit proportion). We found that 2 weeks of exposure to MHW conditions increased resting oxygen consumption (+1°C = 23%, +2°C = 37%), recovery time (+1°C = 62%, +2°C = 77%), EPOC (+1°C = 50%, +2°C = 68%), baseline lactate (+1°C = 27%, +2°C = 28%), post-capture lactate (+1°C = 62%, +2°C = 109%) and haemoglobin levels (+1°C = 13%, +2°C = 28%). This pattern was maintained at 4 weeks of exposure except for post-capture lactate which was reduced (+1°C = −37%, +2°C = 27%). In combination, these results suggest a greater reliance on anaerobic glycolysis to maintain homeostasis in MHW conditions. At 2 weeks post-exposure, when compared to control fish, we found that capture oxygen consumption was increased (+1°C = 25%, +2°C = 26%), recovery rate was increased (+2°C = 38%) and haemoglobin was still higher (+1°C = 15%, +2°C = 21%). These results show that MHW conditions have direct physiological demands on adult coral reef snapper and ecologically relevant residual effects can last for at least 2 weeks post-MHW; however, individuals appear to recover from the negative effects experienced during the MHW. This provides new insight into the effects of MHWs on the physiological performance of coral reef fishes.

## Teaser

Marine heatwaves (MHW) are intensifying, posing a grave threat to coral reefs. We exposed *Lutjanus carponotatus* to MHW conditions (+1°C and + 2°C) for 4 weeks and found increased oxygen consumption and recovery time, among other physiological changes. Interestingly, several effects persisted for at least 2 weeks post-MHW.

## Abbreviations

EPOC: excess post-exercise oxygen consumption
GBR: Great Barrier Reef
MHW: marine heatwave
SST: sea surface temperature

## Introduction

The continued anthropogenic release of greenhouse gases is raising the Earths’ average temperature, along with increasing the frequency and intensity of extreme climatic events (Hoegh-Guldberg *et al*., 2018; Meehl *et al*., 2000; IPCC 2022). The oceans are estimated to have taken up more than 90% of the excess heat in the climate system, leading to unabated ocean warming since the 1970s (Hoegh-Guldberg *et al*., 2018). Both the increasing average temperature of the ocean, and anomalous warming events called marine heatwaves (MHWs), are of concern to marine ecosystems and the biodiversity they support. MHWs are caused by a combination of atmospheric and oceanographic processes with common drivers including persistent high-pressure systems, ocean currents that create a build-up of warm water and air-sea heat flux that transfers atmospheric heat into the sea surface (Hobday *et al*., 2016; Holbrook *et al*., 2019; Gupta *et al.*, 2020). Specifically, MHWs are defined as an anonymously warm event, exceeding the 90^th^ percentile of a 30-year average that lasts for at least 5 days (Hobday *et al*., 2016). Over the last century MHWs have increased in both frequency (34%) and duration (17%) resulting in a 54% increase in the number of MHW days (Oliver *et al*., 2018). The frequency, intensity and duration of MHWs are expected to further increase as anthropogenic climate change continues (Frölicher *et al*., 2018). Consequently, MHWs are considered a more imminent threat to marine organisms than the gradual average increase of sea surface temperature (Oliver, 2019; Smale *et al*., 2019; Guo *et al*., 2022).

Most marine organisms are ectotherms; therefore, an increase in water temperature can result in thermal stress when it exceeds their thermal optima (Somero, 1995; Mora & Maya, 2006; Pinsky *et al*., 2019). Recent MHWs have caused significant mortalities in invertebrates (Garrabou *et al*., 2009), loss of seagrass meadows (Marbà & Duarte, 2010), mass bleaching and mortality in corals (Hughes *et al*., 2017) and are predicted to reduce the biomass of some fish populations (Cheung & Frölicher, 2020). While many marine ecosystems are affected by MHWs (Wernberg *et al*., 2013; Cavole *et al*., 2016; Smale *et al*., 2019), the impacts have been most acutely observed on coral reefs where mass coral bleaching and mortality due to anomalous temperatures have occurred with increasing frequency, magnitude and geographical extent over the past 30 years (Heron *et al*., 2016; Donner *et al*., 2017; Le Nohaïc *et al*., 2017; Hughes *et al*., 2018; Dietzel *et al*., 2020). For example, mass coral bleaching events occurred on the Great Barrier Reef in the summers of 2016 and 2017 where coral mortality was estimated to exceed 50% when averaged over the entire reef (Hughes *et al*., 2018; Stuart-Smith *et al*., 2018). However, documented effects of MHWs on other coral reef organisms are more restricted (Bernal *et al*., 2020). Tropical marine species are predicted to be more sensitive to extreme temperatures than higher latitude species because they have evolved in more thermally stable environments (Tewksbury *et al*., 2008; Sunday *et al*., 2012; Comte & Olden, 2017) and are often living close to their thermal optimum in summer (Rummer *et al*., 2014, Rodgers *et al*., 2018). Yet, the direct effects of MHWs on most coral reef organisms remain unknown (e.g. exceptions; Brown *et al*., 2021, Haguenauer *et al*., 2021; Tran & Johansen, 2023).

Increased water temperature can have broad physiological effects on fish from direct thermodynamic effects on biochemical reaction rates through to changes in whole organism traits such as swimming performance (Clarke & Johnston, 1999; Farrell *et al*., 2009; Little *et al*., 2020). Increasing rates of cellular processes in warmer water results in rising basal metabolic rates (Clarke & Johnston, 1999; Gillooly *et al*., 2001) and elevated energetic costs for physical activities (Johansen & Jones, 2011; Hein & Keirsted, 2012), resulting in increased recovery time (Lee *et al*., 2003; Yanase & Arimoto, 2009) and energetic cost post-exercise (Lee *et al*., 2003; Zeng *et al*., 2010). Furthermore, the additional energy requirement for physical activities may not always be possible through aerobic processes. For instance, the mitochondria, which have a key role in all ATP production, can decrease in efficiency due to enzyme thermal sensitivity, compromising their ability to meet ATP demands when optimal temperatures are surpassed (Brand & Nicholls, 2011). When oxidative metabolism is insufficient, individuals can increase anaerobic metabolism to meet energetic demands (Jacobs, 1986; Omlin & Weber, 2010; Iftikar *et al*., 2014) resulting in costly accumulation of byproducts like blood lactate (Jain & Farrell, 2003; Zakhartsev *et al*., 2004). Water temperature above the thermal optimum can also reduce aerobic capacity (Nilsson *et al*., 2009; Rummer *et al*., 2014; McMahon *et al*., 2020) through an inability of the cardio-vascular system to keep pace with maximum oxygen demands (Farrell *et al*., 2009; Pörtner *et al*., 2017).

While research to date provides some understanding of the likely impacts of elevated temperature on tropical marine fish (Donelson *et al*., 2010; Johansen & Jones, 2011; Rummer *et al*., 2014), much of the work has been designed to explore the effects of longer-term increase in average water temperature (i.e. exposure of mid-century to end of century projections for months to generations) rather than shorter duration warming events and potential recovery trajectories afterwards (Hollowed *et al*., 2013; Lefevre, 2016). However, the rate of change and intensity of MHWs present an acute stress compared to longer warming experiments. Research to date has shown that MHWs in tropical and sub-tropical coral reefs have an average duration of 5–10 and 10–15 days, respectively; however, these durations are expected increase over the current century (Oliver *et al*., 2018). This presents a potential issue as the plasticity of a species to cope with longer, slow changes in temperature may indicate capacity to cope with acute MHW stress. Additionally, current research has shown that coral reef species may not seek thermal refuge during MHWs as previously hypothesized (Haguenauer *et al*., 2021). Recent work has also shown that species may be more sensitive to MHWs over a larger period of the year than previously expected (Tran & Johansen, 2023) which could present unexpected physiological challenges as MHWs increase in duration. The physiological recovery of individuals after MHWs is also an historically overlooked area (Grimmelpont *et al*., 2023); therefore, research encompassing MHWs and post-MHW recovery may provide us with further insight into how marine species will cope with future challenges.

To date, most research on the effects of elevated water temperature on coral reef fishes has focused on smaller bodied, site-attached species, yet the impacts to larger reef fishes may not be easily extrapolated from this. Research into the effects of MHWs on larger predatory fishes, which play an important role in ecosystem function (Ritchie and Johnson, 2009; Hixon, 2015; Hempson *et al*., 2017, 2018), is particularly lacking. Our current knowledge of thermal sensitivity of larger bodied predatory coral reef fish is restricted to a focus on coral trout (*Plectropomus leopardus*), which exhibit high sensitivity to a temperature increases between +1.5 and 4.5°C that results in decreased activity, aerobic scope and survivorship (Johansen *et al*., 2014; Johansen *et al*., 2015; Messmer *et al*., 2017; Pratchett *et al*., 2017). However, this research used temperatures of 30°C and 33°C for a period of 4–6 weeks and consequently may overestimate the impacts of current and imminent MHWs which are on average shorter (5–15 days) in in the species distribution (Oliver *et al*., 2018). We do not yet understand how common MHW conditions may impact reef fish nor how they will recover from these events.

Maximal physiological performance is beneficial for fisheries and bycatch species as the aerobic stress of capture can lead to post-capture mortality, which in recreational catch and release fishing is estimated to be 3–30% depending on the species (Diggles & Ernst, 1997; Frisch & Anderson, 2000; McLeay *et al*., 2002; Sumpton *et al*., 2010). Survival post-capture is important for a number of coral reef fisheries species (e.g. grouper, snapper, trout.) as they are more valuable when sold live (Sadovy *et al.* 2013). Additionally, while the majority of line bycatch is thrown back alive, the sudden and intense stress of capture can have significant physiological impacts and consequently impact survival post-release (Cooke *et al*., 2014; Wilson *et al*., 2014; Raby *et al*., 2018). For example, in a single species fishery, such as coral trout, there are a range of important mesopredator species (e.g. lethrinids, haemulids, lutjanids and serranids) that are caught as bycatch and thrown back (Walton *et al*., 2021). Interestingly, wild populations have also been found to be more susceptible to fishing efforts during MHWs (Brown *et al*., 2021) which could have unforeseen consequences on the health and management of these populations. Further research is essential to understand the impacts of MHWs to larger bodied reef fish including the potential for capture stress associated with fishing during MHWs exacerbating the effects.

Tropical snappers from the family Lutjanidae are among the most abundant mesopredators on coral reefs (Newman *et al*., 1996). They play an important role in ecosystem function (Ritchie and Johnson, 2009; Hixon, 2015; Hempson *et al*., 2018) and are components of both commercial and recreation fishing catches (GBRMPA, 2014). To test the physiological effects of MHWs on a coral reef mesopredatory fish, we subjected adult *Lutjanus carponotatus* (Spanish flag snapper) to two different magnitudes of simulated heatwave conditions, +1°C (29.5°C) and + 2°C (30.5°C) above summer average, for a total of 4 weeks. At 2 weeks of exposure, 4 weeks of exposure and 2 weeks of post-exposure, we measured resting oxygen consumption, capture oxygen consumption, recovery time and associated blood chemistry responses (lactate, glucose, haematocrit and haemoglobin). In a subset of fish not exposed to the MHW treatments, we explored the thermal preference temperature and avoidance temperature to determine how their behavioural thermal optimum range relates to the simulated MHWs temperatures. This experimental design allowed us to measure the physiological effects of MHW conditions on adult Spanish flag snapper, both during and following the period of elevated temperature. If MHW conditions are pushing the species beyond their thermal optimum, we would expect to see signs of increased reliance on anaerobic pathways (e.g. changes in oxygen consumption, recovery times, post exercise oxygen consumption etc.) and biochemical markers (e.g. increased lactate). The repeated measured design allowed determination of whether the magnitude and duration of the warming event had substantive effects on the physiological impacts, as well as whether recovery was possible within 2weeks following a MHW or if there was evidence for lag effects.

## Materials and Methods

### Study species and collection

*Lutjanus carponotatus*, the Spanish flag snapper, is a mesopredator that lives in coral reef habitat throughout the Great Barrier Reef (GBR) and Indo-Pacific region (Allen, 1985). On the GBR, they are often one of the most abundant and widespread species of Lutjanidae on inshore and mid-shelf reefs (Newman *et al*., 1996), and this species is often bycatch from line fishers who are targeting more desirable species such as coral trout. Mature adults for the experiment were collected by professional hook and line collectors from multiple locations in the northern GBR between Cairns and Cape Melville from November 2018 to September 2019. The fish were transferred from holding facilities in Cairns to the Marine and Aquaculture Research Facility at James Cook University, Townsville within 10 days of capture. The average summer sea surface temperature (SST) for the northern GBR, where the fish were collected, is ~28.5°C, with a seasonal range of 24–30°C (AIMS, 2017). This project followed animal ethics guidelines at James Cook University (JCU Animal Ethics No. A2482).

### Aquaria and husbandry

Between six and eight adult fish (28–40 cm standard length) were housed together in twelve 2500 L outdoor tanks (*n* = 80 adults) where they received natural light and lunar events. Each tank was connected to a 10 000 L sump, where water was filtered through a 1 m^3^ sand filter, 25-micron bag filters, and a 400-L protein skimmer. Before being delivered to the tanks the water passed through UV sterilization (~250 mJ cm^3^). Dissolved oxygen was monitored continually with a permanent emersion probe and maintained at 6.1–6.6 mg L^−1^. Salinity was monitored and automatically maintained between 35 and 35.5 ppt by freshwater addition. Adults were fed daily a mixture of Skretting pellets (Spectra SS) and pilchards at ~ 2% body weight. The temperature cycle for all tanks followed a pattern similar to the average monthly temperatures for the northern GBR, where the winter and summer temperatures were 24°C and 28.5°C, respectively.

### Heatwave simulation

Average summer SST is ~28.5°C on the northern GBR, but periods of warmer water, up to 31°C, occur for short periods of time (24–48 hr) in most years (AIMS, 2017). Longer episodes of warmer water, lasting longer than 5 days, are classed as MHWs (Hobday *et al*., 2018). Currently MHWs have been shown to last ~ 5–30 days at sites on the Great Barrier Reef (AIMS, 2017); however, the average duration of MHWs is expected to increase (Oliver *et al*., 2018). To simulate two different intensities of MHW conditions, we chose treatments of +1°C (29.5°C) and + 2°C (30.5°C) for a 4-week period, which is similar in magnitude and duration to recent GBR MHWs (Spinks *et al*., 2019). Control water temperature was maintained at 28.5°C with a heat pump (Toyesi, Titan 20 kW) in a 10 000 L sump. Elevated temperature treatments were achieved with two 5000 L ballast tanks controlled with titanium heat exchangers, one set at 45°C and the other at 13°C. These two water sources were mixed to create accurate delivery water temperatures for the +1°C (29.5°C) and + 2°C (30.5°C) treatments via an automated controller (Innotech, Omni). The temperature treatments were automatically monitored and logged for the duration of the experiment and individual tank temperatures were checked daily. The MHW treatments began in February 2020 after adult fish had spawned. Four tanks were allocated to control, +1°C and + 2°C treatments (12 tanks in total). The warming rate applied was 1°C per 24 hr for both heatwave treatments, which resulted in the +1°C treatment reaching the required temperature in 24 hr and the +2°C treatment reaching required temperature in 48 hr. Once the MHW treatments were achieved, tanks were maintained at that temperature for 4 weeks. Temperature, salinity, pH, and dissolved oxygen were measured, in tanks, daily (Table 1). At the end of 4 weeks, MHW treatments underwent cooling of −1°C per 24 hrs, until they reached the control temperature of 28.5°C (Supplementary Fig. S1).

**Table 1:** Mean (± SD) of experimental seawater chemistry parameters during the marine heatwave (MHW) exposure period (28 days) for *Lutjanus carponotatus*

Treatment	Temperature (°C)	Salinity (ppt)	pH (NBS)	Dissolved oxygen (mg/L)
Control	28.55 ± 0.09	35.41 ± 0.09	8.12 ± 0.02	6.47 ± 0.05
MHW +1 °C	29.54 ± 0.08	35.41 ± 0.09	8.10 ± 0.03	6.45 ± 0.06
MHW +2 °C	30.55 ± 0.08	35.41 ± 0.09	8.11 ± 0.02	6.44 ± 0.05

Tanks were measured daily for temperature, salinity, pH and dissolved oxygen.

### Experimental design

**Figure 1:**
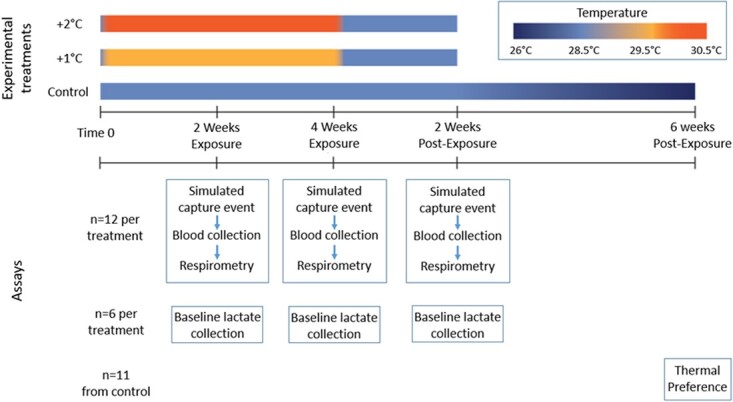
Experimental timeline and assay time points testing the effect of marine heatwaves on adult Spanish flag snapper (*Lutjanus carponotatus*).

A range of experimental assays were conducted over this study at 2 weeks of exposure, 4 weeks of exposure and 2 weeks post-exposure (Fig. 1). The temperature treatment start days for the tanks were staggered (over 5 days) to allow physiological testing to take place on the exact time points for all individuals. At each of these three time points, there were two testing groups per temperature treatment, baseline controls and physiological testing. All fish were fasted for 24 hrs prior to testing. The same individuals were tested at each time point. The first group was baseline controls, which comprised of six fish per treatment, housed in different tanks from individuals used in the physiological testing. Fish were caught directly from their tank and a blood sample was immediately taken. Blood was drawn from the caudal vein (~150 μl blood, < 1% total blood volume) using a hypodermic needle pre-coated in lithium heparin dissolved in fish blood saline (21-G, 1 ml syringe). An aliquot of 15 μl was used to determine baseline blood lactate (mmol per L) with the Accutrend Plus multi test meter (Roche Diagnostics Australia). These individuals were not used in any other subsequent assays for that time point. The second group, physiological testing, was comprised of 12 individuals from each of the three temperature treatments. This testing group was put through a simulated capture event, which was immediately followed by blood collection (<1% total blood volume), followed by respirometry to estimate oxygen consumption (MO_2_) over the subsequent ~ 20 h (details below). Following the 6-week MHW exposure period experimental temperatures was brought down from 28.5°C to 26°C, over a 4-week period, at which point 11 individuals from the control treatment were used to determine preferential temperature.

**Figure 2:**
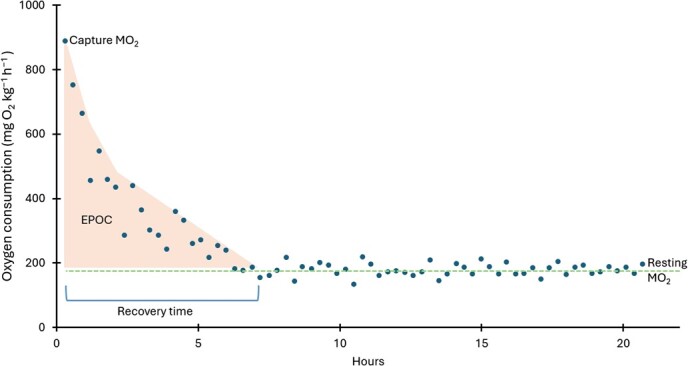
Aerobic metrics measured in this experiment. The figure shows MO_2_ data from a single individual. During the assay we measured the capture MO_2_ (highest MO_2_) and allowed the individual to return to resting MO_2_. Recovery time was also measured. Excessive post-exercise oxygen consumption (EPOC), illustrated by shaded, triangular area, was then calculated from these metrics (as described in methods section).

### Simulated capture event

This assay was designed to emulate a natural aerobic challenge more representative of the events encountered by wild populations, diverging from conventional methodologies historically employed. To achieve this, we simulated a hook and line capture event using a 90-L swim tunnel (Loligo, fitted with a TechTop 1 hp, 2880 rpm motor) to elicit sudden burst swimming representing an aerobic challenge. Twelve fish from each treatment (36 fish total, standard length = 310 mm ± 31 mm, weight = 588 g ± 87 g, mean ± SE) were placed in the swimming chamber at a slow water speed of 5 cm/s. Once the individual had orientated into the water flow (~5–10 s on average) the water speed was immediately increased to full speed (from 5 cm s^−1^ to 200 cm s^−1^ in 5 s), equating to ~6–7 body lengths per second. This protocol elicited bursting and erratic swimming in individuals which represent a significant aerobic challenge. This was sustained for 60 s after which point the water flow was stopped and the fish removed. As the species are typically found in shallow reef environments (<20 m depth) a fishing capture event would typically be between 30 and 60 seconds (information from line fisherman that collected broodstock; Cooke *et al*., 2014; Raby *et al*., 2018) and the species is known to swim intensely for the duration of the event. This simulation is intended to be an alternative, more realistic representative measurement of sudden and exertive aerobic activity for this species than the traditional chase methods.

### Blood collection and analysis

Blood was drawn from all fish following the capture stress assay, prior to respirometry testing (Wood and Munger, 1994). A minimal amount of blood was drawn from the caudal vein (~150 μl blood, < 1% total blood volume) using a hypodermic needle pre-coated in lithium heparin (21-G, 1 ml syringe). The blood drawing site was cleaned with betadine wipes and the procedure took no longer than 60 s. Each blood sample was immediately used to measure the concentration of lactate (mmol per L^−1^) and glucose (mg L^−1^) from 15 μl samples using the Accutrend Plus multi test meter (Roche Diagnostics Australia). Haemoglobin was then calculated by Drabkin’s method (Drabkin and Austin, 1935; Balasubramaniam and Malathi, 1992) using a spectrophotometer (Thermo Scientific, Spectronic 200) to calculate the haemoglobin (g L^−1^). Finally, haematocrit proportion was calculated by centrifuging three micro capillary tubes with ~ 30 μl of blood for 3 minutes, then averaging the proportion of packed red blood cells of the three tubes.

### Intermittent flow respirometry

Aerobic performance was measured using intermittent flow respirometry and fish were fasted for 24 h before respirometry (Clark *et al*., 2013; Svendsen *et al*., 2016) (*n* = 12 per treatment). Fish were tested at their respective treatments at 2 and 4 weeks of exposure and then all individuals were tested at control temperature 2-week post-exposure (Fig. 1; control: 28.5°C, +1°C: 29.5°C and + 2°C: 30.5°C). Respirometry was conducted in purpose-built intermittent flow respirometry chambers (35.5 L per closed system), submerged in aquaria within the individual’s respective experimental treatment water. Submersible pumps fitted to each chamber supplied a continuous water flow from the surrounding water bath through the chambers. Activity was reduced in the respiration chambers by using appropriately sized chambers to minimise movement and by shading each chamber from visual simulants. A purpose-built python program, AquaResp v3.0, was used to control the measurement cycle timing. This consisted of a 10-minute measurement period, 8-minute flushing period, and a 2-minute wait period, which was repeated over 24 h trial duration. The O_2_ consumption rates were measured during the intervals of interrupted water flow with a Firesting Optical Oxygen Meter (Pyro Science e. K., Aachen, Germany), which the AquaResp program recorded during the measurement periods. The entire measurement period was used to calculate MO_2_ provided that the slope *R*^2^ was > 0.90 (Fig. 2). Over 93% of measured slopes across all treatments were above this threshold. The 7% of slopes that were under 0.90 *R*^2^ were not used. Fish were immediately placed into respirometry chambers following the capture assay and 1 minute air exposure where blood was drawn (<1% total volume), and the fish were weighed. Measurement started once the chamber was closed allowing for capture MO_2_ to be measured. Fish then remained in the chambers while recovering back to resting MO_2_ over 20 hours.

Capture MO_2_ and resting MO_2_ of individuals was calculated in mg O_2_ kg^−1^ h^−1^ using the equation:

 where *K* is the linear rate of decline (kPah^−1^) in the oxygen content over time (h) in the respirometer; *V* is the volume of the respirometer in L, which is adjusted for the volume of the fish; β is the solubility of oxygen in water at a specific temperature and salinity (mg O_2_ L^−1^ kPa^−1^) and *M* is the body mass of the fish (kg). Blank measurements were taken for each chamber at the start and end of each trail to calculate any background respiration.

Background respiration did not exceed 5 mg O_2_ kg^−1^ h^−1^ in any trial. Linear regressions were then used to calculate background respiration over the trail, which was used to adjust the MO_2_ measurements for each fish. Capture MO_2_ was determined from the first O_2_ reading, directly after the capture assay was completed. Resting MO_2_ was determined by using the mean of the lowest normal distribution for MO_2_ values (Behrens and Steffensen, 2007; Chabot *et al*., 2016).

Recovery time was determined by amount of time it took for oxygen consumption to decline from capture MO_2_ to the intercept of the resting MO_2_ (Fig. 2). Resting MO_2_ was used as a proxy for resting metabolic rate and capture MO_2_ was used to estimate the metabolic demands of the capture event.

Excessive post-exercise oxygen consumption (EPOC) was calculated as the area below the slope between capture MO_2_ and resting MO_2_ for each fish (Fig. 2). Specifically, the area created between all consecutive measurement cycles of 20 minutes was calculated as an irregular quadrilateral, and then summed to get the EPOC value (mg O_2_ kg^−1^) for each fish.

Finally, recovery rate was calculated by dividing an individual’s EPOC (mg O_2_ kg^−1^) by their recovery time (minutes) and then multiplying by 60 (minutes) to standardise the recovery rate as (mg O_2_ kg^−1^ h^−1^).

### Temperature preference

Thermal preference is expected to indicate the optimal temperature for physiological functions (Martin and Huey, 2008; Angilletta, 2009), and in some species has been correlated to thermal optima for growth (Killen, 2014). To determine the temperature preference in this population of *L. carponotatus* and how this related to the physiological thermal sensitivity to MHWs, we used a custom-built acrylic shuttle box arena (Chambers = diameter 80 cm/total arena volume of 330 L) in conjunction with commercial tracking software and controllers (Loligo, ShuttleSoft), to conduct a preferential temperature test (Killen, 2014). All individuals were held at 26°C before the experiment began as it is the approximate mid-point of this species temperature range. The individuals in these assays were not put through the MHW treatments. The experiment was automated to determine the preferred temperature and avoidance temperature of adult *L. carponotatus*. Before the test an individual (from control treatment only; Fig. 1) was placed in the arena at 1600, with one chamber set at 25°C and the other at 27°C (static mode). Fish were then allowed to habituate to the arena overnight until the preference trial started at 0800. The trial was run in dynamic mode where the position of the fish in terms of chamber (cool or warm) determined whether the system heated or cooled at a rate no faster than 2°C per hour, while maintaining 2°C differential. For all fish, the automated system started by increasing the temperature of both sides as all fish were found within the warm chamber. Avoidance temperature was determined once the individual left the warmer side of the arena for sufficient time to start cooling both sides. The preferential temperature was determined when the individual positioned itself in the thoroughfare between the two sides or swam continuously between both sides, thereby stopping any further shift in temperature.

### Statistical analysis

Linear mixed effects (LME) models with Gaussian distributions were used for all measured physiological responses to identify significant differences between treatments, except for haematocrit, which was analysed with a generalized linear mixed model (GLMM) with a Gaussian distribution. In all LMEs, and in the GLMM for haematocrit, temperature and exposure time (2 weeks, 4 weeks and 2 weeks post-MHW) were fixed factors. In addition, interactions between temperature and exposure time were included in the models to assess their combined effects on the physiological responses. Tank, testing day and individual ID were used as random effects. Following the construction of the linear mixed-effects model, the significance of the main effects and their interaction was evaluated using a Type III ANOVA with degrees of freedom calculated using the restricted maximum likelihood (REML) method. Following this, Tukey’s post hoc tests were conducted on any models with significant effects. All analyses were conducted in R (R Core Team, 2014) using the LME4 and GLMM packages (Bates *et al*., 2015). Tukey’s post hoc tests were conducted on significant factors while estimated marginal means tests, adjusted with Tukey’s (emmeans). All models met the assumptions of the relevant tests. This was confirmed by accessing the residuals, goodness of fit and checking dispersion.

## Results

### Aerobic performance

**Figure 3:**
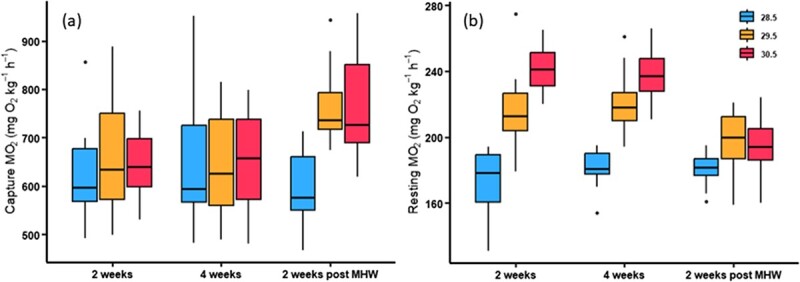
Box and whisker plots show capture oxygen consumption (a), and resting oxygen consumption (b) of adult *Lutjanus carponotatus* under ambient (28.5°C) conditions and two marine heatwave treatments of +1°C (29.5°C) and + 2°C (30.5°C). Individuals were tested at 2 weeks exposure, 4 weeks exposure and 2 weeks post-exposure.

The capture MO_2_ of individuals was significantly affected by MHW treatments (*F*_2,97_ = 6.01, *P* = 0.004), exposure time (*F*_2,97_ = 6.36, *P* = 0.003) and the difference between temperature treatments was not consistent across time points (Treatment*Exposure Time: *F*_2,97_ = 4.65, *P* = 0.002; Fig. 3a). Specifically, capture MO_2_ was similar across treatments for both 2 and 4 weeks of exposure at ~ 640 mg O_2_ kg h^−1^ (all post hocs *P* > 0.05; Supplementary Table S1). However, at 2 weeks post-exposure capture MO_2_ was increased by 25% in fish that had experienced the +1°C treatment (~764 mg O_2_ kg h^−1^; *t* = −4.68, *P* < 0.001) and the +2°C treatment (~765 mg O_2_ kg h^−1^; *t* = −4.70, *P* < 0.001) compared to control fish (~594 mg O_2_ kg h^−1^; Fig. 3a).

Resting MO_2_ was significantly higher in the MHW treatments (*F*_2,97_ = 66.52, *P* < 0.001) compared to the controls, varied between sampling time points (*F*_2,97_ = 18.15, *P* < 0.001), and there was an interaction between temperature treatment and sampling time point (*F*_4,97_ = 7.92, *P* < 0.001; Fig. 3b). In fish exposed to the +1°C MHW treatment resting MO_2_ increased on average by 22–23% at 2- (220 mg O_2_ kg h^−1^; *t* = −5.96, *P* < 0.001) and 4- (216 mg O_2_ kg h^−1^; *t* = −5.63, *P* < 0.001) weeks of exposure compared to control fish (180 mg O_2_ kg h^−1^). A similar pattern was seen in the +2°C MHW treatment where resting MO_2_ was significantly increased on average by 31–33% after 2- (240 mg O_2_ kg h^−1^; *t* = −9.44, *P* < 0.001) and 4- (237 mg O_2_ kg h^−1^; *t* = −7.99, *P* < 0.001) weeks of exposure compared to control fish. At 2 weeks post-exposure, resting MO_2_ in both MHW treatments was lower than during the warming exposure (12% and 23% reduction in +1°C and + 2°C fish, respectively) and was no longer higher than in control fish (+1°C: *t* = −2.32, *P* = 0.337; +2°C: *t* = −1.95, *P* = 0.583). The resting MO_2_ of control fish remained at ~180 mg O_2_ kg h^−1^ throughout the 6-week experiment (Fig. 3b).

**Figure 4:**
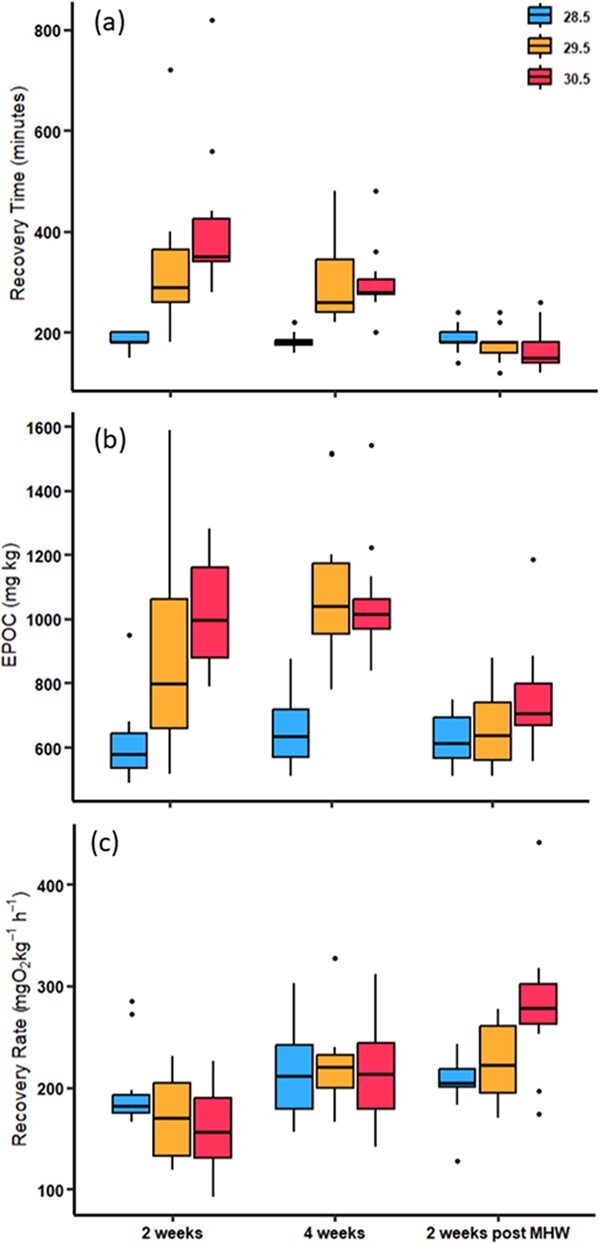
Box and whisker plots show recovery time (a), EPOC (b), and recovery rate (c) of adult *Lutjanus carponotatus* under ambient (28.5°C) conditions and two marine heatwave treatments of +1°C (29.5°C) and + 2°C (30.5°C). Individuals were tested at 2 weeks of exposure, 4 weeks of exposure and 2 weeks post-exposure.

Recovery time following the simulated capture event was significantly higher in MHW treatments (*F*_2,97_ = 24.27, *P* < 0.001), differed between sampling time points (*F*_2,97_ = 33.71, *P* < 0.001) and there was a significant interaction between temperature treatments and time point (*F*_2,97_ = 9.56, *P* < 0.001; Fig. 4a). In the +1°C MHW treatment, recovery time at 2 weeks of exposure was approximately 140 minutes longer (77% increase; *t* = −5.74, *P* < 0.001) than control fish and remained significantly longer at 4 weeks of exposure (120 minutes longer, 66% increase; *t* = −4.90, *P* < 0.001). For fish in the +2°C MHW treatment, recovery took approximately 170 minutes longer at 2 weeks of exposure (94% increase; *t* = −6.95, *P* < 0.001) and 120 minutes longer at 4 weeks of exposure (64% increase; *t* = −4.70, *P* < 0.001) than control fish (Fig. 4a). Additionally, the +2°C MHW treatment fish showed a significant reduction (30%) in recovery time at 4 weeks of exposure (*t* = 3.59, *P* = 0.015). At 2 weeks post-exposure, the recovery time for fish that had been exposed to either MHW treatment took on average 180 minutes, which was significantly lower than during the MHW phase (all > 300 minutes, *P* < 0.001; Supplementary Table S2). Post-exposure recovery times were comparable to the control fish (all *P* > 0.05). Recovery time was similar between the two MHW treatments at each of the exposure timings (all post hocs *P* > 0.05; Supplementary Table S2).

EPOC was significantly higher in MHW treatment fish (*F*_2,97_ = 33.26, *P* < 0.001; Fig. 4b), and this pattern changed across exposure time (*F*_2,97_ = 19.99, *P* < 0.001). At 2 weeks of exposure, the EPOC of MHW fish was found to be on average 50% higher than control in the +1°C treatment (*t* = −4.28, *P* < 0.001) and 68% higher in the +2°C treatment (*t* = −5.89, *P* < 0.001). At 4 weeks of exposure, the EPOC of +1°C and + 2°C MHW treatment fish was found to be higher than control fish by 67% (*t* = −6.99, *P* < 0.001) and 61% (*t* = −6.33, *P* = 0.015), respectively. While there was a trend for EPOC to still be higher than control fish at 2 weeks post-exposure, +1°C (5%) and + 2°C (18%), MHW treated fish were no longer significantly different from control (*P* > 0.05, Supplementary Table S3). There was no significant difference between the EPOC of fish from +1°C and + 2°C MHW treatments at any sampling time point (all post hoc *P* > 0.05).

Temperature and exposure time point had an interactive effect on recovery rate (*F*_2,97_ = 5.59, *P* < 0.001; Fig. 4c). Recovery rates were not significantly different between treatments at 2 weeks of exposure (mean control = 197, +1°C = 171, +2°C = 158 mg O_2_ kg^−^1 h^−1^) (Supplementary Table S4). However, at 4 weeks of exposure all treatments were significantly higher when compared to their respective treatments at 2 weeks of exposure by 37–40% (Supplementary Table S4). At 2 weeks post-exposure fish from +2°C treatment had the highest recovery rate (281 mg O_2_ kg^−1^ h^−1^) compared all other treatments across time points (ranging from 26 to 77%) with the exception of fish from +1°C treatment at 2 weeks post-exposure (Supplementary Table S4).

### Blood parameters

**Figure 5:**
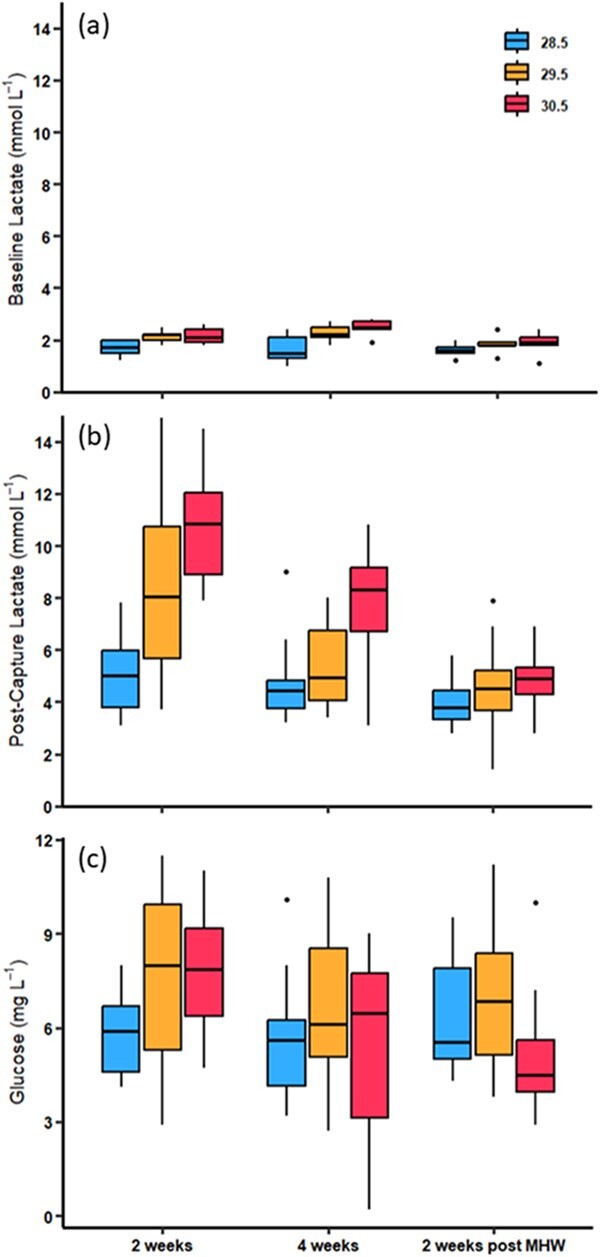
Box and whisker plots show baseline lactate (a), post-capture lactate (b), and glucose (c) of adult *Lutjanus carponotatus* under ambient (28.5°C) conditions and two marine heatwave treatments of +1°C (29.5°C) and + 2°C (30.5°C). Individuals were tested at 2 weeks of exposure, 4 weeks of exposure and 2 weeks post-exposure.

Baseline blood lactate was significantly affected by MHW treatments (*F*_2,34_ = 7.64, *P* = 0.002) and sampling time point (*F*_2,34_ = 3.32, *P* = 0.048; Fig. 5a), but there was no interaction between treatments and sampling time point (*F*_4,34_ = 0.65, *P* = 0.628). Baseline lactate in control fish was 1.68 mmol L^−1^ at 2 weeks of exposure (Fig. 5a). Baseline blood lactate was 27% and 28% higher than control levels in the +1°C and + 2°C MHW treatments respectively. At 4 weeks of exposure, the baseline blood lactate levels in the MHW treatments were 36% (+1°C) and 48% (+2°C) higher than in the control fish (1.66 μmol ml^3^). At 2 weeks post-exposure, the blood lactate of MHW treated fish had reduced to levels closer to control fish, albeit still ~ 15% higher (Fig. 5a). This pattern resulted in significant differences in blood lactate between both +1°C and + 2°C MHW treatments compared with control fish (control vs +1°C: *t* = −3.07, *P* = 0.012; control vs +2°C: *t* = −3.63, *P* = 0.003) and between 4 weeks of exposure and 2-week post-exposure (*t* = 2.55, *P* = 0.04).

Post-capture blood lactate was significantly affected by MHW treatment (*F*_2,97_ = 26.17, *P* < 0.001), exposure sampling time point (*F*_2,97_ = 32.59, *P* < 0.001) and the interaction between temperature and sampling time point (*F*_4,97_ = 5.15, *P* < 0.001; Fig. 5b). Specifically, post-capture lactate was higher at 2 weeks of exposure in fish from both the +1°C (8.3 mmol L^−1^, 62% higher; *t* = −4.12, *P* = 0.002) and the +2°C MHW treatment (10.7 mmol L^−1^, 109% higher; *t* = −7.26, *P* < 0.001) compared with control fish (5.1 mmol L^−1^). Post-capture blood lactate was significantly lower after 4 weeks compared with the 2-week sampling point for both the +1°C (5.29 mmol L^−1^, 37% reduction; 2 weeks vs 4 weeks: *t* = 3.92, *P* = 0.005) and + 2°C MHW treatments (7.84 mmol L^−1^, 27% reduction; 2 weeks vs 4 weeks: *t* = 3.76, *P* = 0.009; Fig. 5b). This resulted in only the +2°C MHW treatment fish having higher post-capture lactate than control (*t* = −4.03, *P* = 0.003) and + 1°C MHW fish (*t* = −3.29, *P* = 0.036) at the 4-week sampling point. At 2 weeks post-exposure, fish from the +2°C MHW treatment had reduced post-capture lactate levels compared with the 4-week sampling point (38% reduction; *t* = 3.78, *P* = 0.008), such that no treatments were different from each other at 2 weeks post-exposure (control vs +1°C: *t* = −0.76, *P* = 0.976; control vs +2°C: *t* = −1.22, *P* = 0.949; +1°C vs +2°C: *t* = −0.45, *P* = 0.999). Blood glucose levels were not significantly affected by MHW treatments (*F*_2,97_ = 2.90, *P* = 0.06), sampling time point (*F*_2,97_ = 2.51, *P* = 0.09), and there was no interaction between treatment and time point (*F*_4,97_ = 1.83, *P* = 0.129; Fig. 5c).

**Figure 6:**
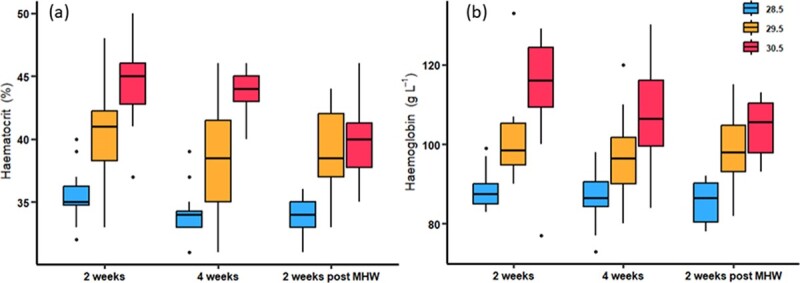
Box and whisker plots show haematocrit (a) and Haemoglobin (b) of adult *Lutjanus carponotatus* under ambient (28.5°C) conditions and two marine heatwave treatments of +1°C (29.5°C) and + 2°C (30.5°C). Individuals were tested at 2 weeks of exposure, 4 weeks of exposure and 2 weeks post-exposure.

MHW treatments significantly increased haemoglobin (*F*_2,97_ = 43.36, *P* < 0.001) and haematocrit (*X*^2^ = 139.00, df = 2,97, *P* < 0.001; Fig. 6a). Haematocrit and haemoglobin concentration was highest in the +2°C treatment (Haematocrit: 44.5%, Haemoglobin: 114 g L^−1^), followed by the +1°C (Haematocrit: 40.3%, Haemoglobin: 101 g L^−1^) and lowest in control fish (Haematocrit: 35.5%, Haemoglobin: 89 g L^−1^; all *P* < 0.05). On average, there was ~ 13% more red blood cells for every 1°C temperature increase. Both haematocrit and haemoglobin exhibited a pattern of generally decreasing with time, however, this was only significant for haematocrit (*X*^2^ = 13.44, df = 2,97, *P* = 0.001; Fig. 6a) and not for haemoglobin (F_2,97_ = 2.89, *P* = 0.06; Fig. 6b). Furthermore, this decrease in heamatocrit was only significant when comparing 2 weeks of exposure and 2 weeks post-exposure (*t* = 3.67, *P* = 0.001, Fig. 6a).

### Temperature preference

The preferred temperature of *L. carponotatus* was 29.81°C ± 0.25 (mean ± SE) (range 28–31.2°C). The avoidance temperature was ~2°C warmer than the preferred temperature at 31.93°C ± 0.22 (range 29.5°C–33.1°C). All individuals started the trial by increasing the arena temperature, by staying in the warmer side, until they reached their avoidance temperature at which point, they moved to the cooler side and then swam evenly between the warm and cool side to stop the shifting of arena temperatures.

## Discussion

The increased frequency and intensity of MHWs pose a significant threat to marine organisms, especially those adapted to stable thermal environments like coral reefs. Our study revealed distinct physiological responses in adult *L. carponotatus* under simulated MHW conditions, at modest temperature increases of +1 to +2°C above the summer average. During MHWs, fish exhibited higher metabolic activity and signs of elevated stress, yet mostly recovered within 2 weeks post-exposure. The heightened basal cellular costs (resting MO_2_), prolonged recovery time after capture, and elevated blood lactate indicate negative physiological impacts of MHW conditions. Depending on MHW duration in nature, this could potentially reduce body condition and hinder the ability to escape predators or find prey (Killen *et al*., 2015; von Biela *et al*., 2019). Interestingly, haemoglobin and haematocrit remained elevated at 2 weeks post-exposure, and capture MO_2_ significantly increased, suggesting a shift in relative aerobic and anaerobic energy production, corroborated by post-capture lactate levels.

Thermal stress manifested in multiple physiological measures during MHW exposure. Resting MO_2_, recovery time, EPOC and baseline blood lactate levels all significantly increased in both MHW treatments over the 4-week exposure period but showed recovery within 2-weeks after returning to control temperature (i.e. post-exposure). Resting MO_2_ and basic metabolic costs generally rise by ~2–14% (Q10: 1.48–3.71) with every degree of warming during summer for tropical coral reef fish (Nilsson *et al*., 2009; Johansen and Jones, 2011; Rummer *et al*., 2014; Messmer *et al*., 2017; Pratchett *et al*., 2017). In *L. carponotatus*, resting MO_2_ increased by 10–20% per degree Celsius (Q10: 2.86–7.93), indicating a high degree of thermal sensitivity compared to other coral reef species, which is similar to the larger-bodied mesopredator, coral trout (10–14% increase per degree celsius) (Messmer *et al*., 2017; Pratchett *et al*., 2017). Additionally, we observed prolonged recovery time for fish to reach resting MO_2_ after simulated capture stress, with individuals taking ~2 hours longer in both +1°C and + 2°C MHW conditions. This extended period not only incurs elevated metabolic rate costs but also exposes them to increased predation risk due to reduced aerobic escape capacity (Killen *et al*., 2015). Fish from both MHW treatments also exhibited higher baseline lactate levels (27–48% higher than controls), indicating a greater reliance on anaerobic glycolysis for energy production. This is perhaps due to thermal effects on mitochondrial efficiency or simply an inability to meet required energy aerobically as resting MO_2_ also increased (Jacobs, 1986; Omlin and Weber, 2010; Iftikar *et al*., 2014). Furthermore, there was a trend of accumulating blood lactate, and potentially stress, over time in MHW-exposed fish, as indicated by a general increase (15–20%) in lactate between the 2- and 4-week exposure periods. Nevertheless, these fish were capable of processing lactate once temperatures returned to normal.

During exercise, blood lactate levels can rapidly increase to supplement energy demand for swimming (Jones, 1982; Weber *et al*., 2016). Under MHW conditions, blood lactate levels increased significantly (67–109%) due to simulated capture stress compared to the baseline increase (27%) at the same MHW exposure time point. Since capture MO_2_ remained consistent across all treatments during MHW exposure, it is likely that individuals in the elevated MHW treatments lacked sufficient aerobic capacity, leading to increased anaerobic energy production as compensation (Drucker and Jensen, 1996; Svendsen *et al*., 2010). The additional lactate may have contributed to the observed longer recovery times as aerobic metabolism remained elevated to oxidize lactate from the blood (Wells *et al*., 2009; Ohlendieck, 2010). Interestingly, when comparing baseline and post-capture lactate, distinct patterns emerged over time. Baseline lactate tended to increase at the 2-week exposure mark and remained high at 4-weeks, whereas post-capture lactate decreased between the 2- and 4-week exposure periods. This suggests that no physiological mechanisms were induced to offset the fundamental cellular costs of functioning under elevated conditions. However, it is plausible that other unmeasured physiological mechanisms were initiated, resulting in a reduction of lactate following the capture event (e.g. upregulation of lactate dehydrogenase; Larios-Soriano *et al*., 2020).

Haemoglobin and haematocrit levels were elevated during MHW treatments, with residual effects observed post-exposure. Increased production of red blood cells (RBCs), indicated by higher haematocrit, may have been prompted to meet the elevated respiration and energy demands caused by higher temperatures (Gillooly and Zenil-Ferguson, 2014). Additional RBCs would enhance oxygen transport capacity and gill diffusion efficiency in lower dissolved oxygen concentrations at higher temperatures (Wells and Baldwin, 1990; Gallaugher and Farrell, 1998). We might expect a corresponding shift in aerobic performance, including increased resting and capture respiration. Resting MO_2_ followed expectations, showing increased demand during MHW, however, MO_2_ during simulated capture stress did not increase, despite the presence of additional RBCs. While capture swimming costs may not have risen in MHW treatments (i.e. if fish were within their thermal optimal range), higher lactate levels and extended recovery time indicate increased energy demand for swimming in MHWs. Therefore, swimming physiology factors, rather than oxygen delivery, likely limited capture MO_2_ (Pörtner, 2010; Pörtner *et al*., 2017). For example, capture MO_2_ may represent the maximum capacity of aerobic swimming (Schulte, 2015), which remained consistent across the three treatments during MHW. Alternatively, our measurements may represent the limit of aerobically generated energy (i.e. Krebs cycle: Krebs, 1950). While the specific physiological mechanism remains unclear in this study, it suggests the existence of an aerobic capacity limit is unaffected by MHW treatment.

While the additional RBCs did not alter capture MO_2_ during MHW conditions, they may have had an effect once temperature returned to normal in the 2 weeks following. Capture MO_2_ was 25% higher than control, and higher than these fish during the MHW, in both +1°C and + 2°C MHW treatments at 2-week post-exposure while haemoglobin and haematocrit remained elevated. The lifespan of a RBC is thought to be ~ 60–120 days (Franco, 2012; Shrestha *et al*., 2016) and if the initial temperature increase induced the production of additional RBCs, they would not be destroyed or discarded if healthy. Thus, the elevated proportion of RBCs (haematocrit) post-exposure are likely to be a legacy of MHW exposure rather than an active response. Interestingly, alongside this elevated aerobic response, higher lactate levels and recovery time returned to control levels in MHW fish post-exposure. This suggests that the relative production of aerobic to anaerobic energy during swimming capture has shifted, and perhaps the increase in RBC is assisting the increase of aerobic energy production (Mairbäurl, 2013; Saunders *et al*., 2013). This point is strengthened by the reduced EPOC found at 2 weeks post-exposure as while there is higher aerobic demand seen during the capture event there is not a significantly higher debt post exercise, which may indicate reduced reliance on anaerobic energy.

Although we observed some persistence of physiological effects post-MHW exposure, the fish generally showed limited stress and costs 2 weeks post-MHWs. The only physiological attribute that suggested a continuing cost was resting MO_2_ (~9% higher), however, this was not statistically significant. Baseline lactate and recovery time, while significantly higher during the MHW exposure phase, both returned to control levels within 2 weeks of fish returning to control conditions. There may have been other energetic or physiological costs associated with MHWs that were not measured here, such as the release and replacement of hormones (Iwama *et al*., 1998; Iwama *et al*., 1999; Alfonso *et al*., 2020), production of proteins (Foster *et al*., 1992; Jonassen *et al*., 1999; Larios-Soriano *et al*., 2020; Johansen *et al*., 2021), and cell repair from oxidative stress (Lepock, 2005; Lushchak and Bagnyukova, 2006; Madeira *et al*., 2013; Birnie-Gauvin *et al*., 2017) that may still impose an energetic cost on fish following a MHW. It is also possible that we would have observed costs in the metrics we investigated had we measured closer to the end of exposure (e.g. 1-week post-exposure). However, it is encouraging that while *L. carponotatus* is sensitive to MHWs, there is a relatively rapid recovery of the physiological system within 2 weeks. This suggests that MHWs similar to the length and duration used in this study may not have significant, longer-lasting effects on these fish.

Physiological changes measured in this study are expected to be a result of thermal effect on molecular processes, cellular stress response and cellular homeostasis response (Kültz, 2003; Kültz, 2004). When stressful conditions persist, genes may be up or down regulated to adjust cellular and whole organism phenotype in response to the altered environmental conditions. Only one study so far has investigated the molecular response of coral reef fish (damselfish and cardinalfish) to a natural MHW (Bernal *et al*., 2020). Interestingly, Bernal *et al*. (2020) found that during a MHW fish exhibited gene expression changes that related to metabolic processes, cell damage and cell repair. At the peak of the MHW gene expression differences were associated with processes including mitochondrial activity, adenosine triphosphate activity and cholesterol and fatty acid metabolism, which may indicate genomic level effects to the higher level metabolic processes in the present study.

The average preference temperature of *L. carponotatus* (29.8°C) resembled the +1°C MHW treatment (29.5°C) used in this study, where stress indicators (baseline lactate) and increased energetic costs (resting MO_2_ and recovery time) were observed. Similar temperatures have been recorded for other reef fish species from the GBR, such as the five-lined cardinalfish (29.5°C, Nay *et al*., 2015) and the blue green damselfish (28.9°C, Habary *et al*., 2017). It is possible that other unmeasured physiological responses, such as reproduction and enzyme activity, may be enhanced at warmer conditions, optimizing the overall fitness of this species at 29.8°C. However, preference temperature may not always align with natural selection due to ecological factors (territory, shelter, food) influencing body temperature (Hugie and Dill, 1994; Lindberg *et al*., 2006; Eurich *et al*., 2018). This preferred temperature suggests that shorter warming events (<2 weeks) may not pose a physiological challenge, but further investigations are needed. The avoidance temperature for *L. carponotatus* was 3.4°C above the summer average and 1.1°C above preference, indicating a narrow thermal window. Concerningly, GBR reefs have already experienced temperatures exceeding this avoidance threshold during MHWs in 2016, 2017 and 2020 (AIMS, 2017; Hughes *et al*., 2018; Huang *et al*., 2024), suggesting some reefs have already been close to the species’ thermal limits. While our experimental evidence that fish will actively avoid warm temperature is perhaps encouraging for survival in nature, thermal refuges may not be available or used in nature. *L. carponotatus* has evolved in shallow tropical reefs and individuals may simply remain in their established home ranges during MHWs and endure the physiological challenges. The recovery speed from MHWs we observed may support this hypothesis, similar to the strategy observed in the coral reef mesopredator, *Plectropomus leopardus*, which reduced activity but increased feeding rate as temperatures rose above the summer average (Scott *et al*., 2019). Regardless of the strategy employed, managing the energetic requirements during MHWs in nature could be challenging.

Interestingly, the physiological effects of the two different magnitudes of simulated MHW (+1°C and + 2°C) were often similar. The temperature increase of +1°C elicited a significant response in baseline lactate, post-capture lactate (exception 4-weeks of exposure), resting metabolic rate, and recovery time, which was not proportionally increased further at +2°C (i.e. there was not an additive effect of each 1°C temperature increase in the MHW treatments). This suggests that not all physiological processes will be affected linearly by MHWs which supports the hypothesis that tropical species are sensitive to relatively small temperature increases and are living close to their thermal optimum during summer (Tewksbury *et al*., 2008; Sunday *et al*., 2012; Rummer *et al*., 2014; Comte and Olden, 2017; Rodgers *et al*., 2018). The complexity of these various thermal physiological responses indicates the importance of understanding a range of physiological traits when investigating the effects of future MHWs on wild populations, as no single metric is sufficient to comprehend the whole animal physiological response to elevated temperature.

Due to the increasing frequency and intensity of MHWs, there is an immediate need to understand the sensitivity of organisms both during and following MHWs. This study shows that while short-term (2–4 weeks) exposure to MHWs has significant effects on the physiological response of a coral reef snapper there is a relatively rapid restoration to baseline levels post-exposure within 2 weeks. One point to consider about these findings is that the individuals used in this study were all caught by hook and line. This could introduce bias towards specific phenotypes (e.g. bolder, more active, etc.) potentially leading to over- or under-representation of effects within the wild population. While there is limited literature on this, if phenotypic selection has occurred, we might expect that these bolder individuals will have higher metabolic rates (Fu *et al*., 2021) and are the most thermally sensitive (Norin *et al*. 2024). Nevertheless, while fish appear able to alter their physiological processes to cope with MHWs, the elevated resting metabolic rate suggests that fish still need to obtain about 20% more energetic resources to sustain basic maintenance during MHWs. For mesopredators, this would ultimately mean increased predation of smaller reef organisms, which could flow on to affect abundance and assemblage composition of lower trophic levels (Boaden and Kingsford, 2015), or conversely a decline in condition if energy requirements are not met (Hempson *et al*., 2018). If not able to be met with intake, these increased energy demands could mean a trade-off by decreasing other activities like growth or reproduction, which might influence population dynamics of *L. carponotatus*. Additionally, the stress on individuals during MHWs may be compounded by fishing pressure. As MHWs induce a range of physiological responses in individuals, additional pressure from fishing activities may exacerbate the physical strain on this species. Catch-and-release fishing, whether recreational or commercial bycatch, during MHWs may significantly impact individuals’ health and survival rates, and therefore affect a population’s overall health and persistence. In planning conservation measures, it would be prudent to consider implementing fishing restrictions during heatwaves to alleviate these stress effects. Further research into how the duration and intensity of MHWs affect the physiology of a range of coral reef fishes would help us identify the physiological limits, processes and costs that future MHWs may impose on these important ecosystems and food resources globally.

## Supplementary Material

Web_Material_coae060

## Data Availability

The data sets are available on JCU’s Tropical Data hub at https://doi.org/10.25903/c10b-an63
