# Supplementary material for: The effects of marine heatwaves on a coral reef snapper: insights into aerobic and anaerobic physiology and recovery
Source: Conserv Physiol. 2024 Aug 21;12(1):coae060. doi: 10.1093/conphys/coae060 (PMC11793158; doi:10.1093/conphys/coae060)
Supplement: Web_Material_coae060 [file web_material_coae060.zip › Supplementary tables.pdf]

Table S1. Tukey's post-hoc test on the capture MO_2 of *Lutjanus carponotatus*. Adults were held either control (28.5°C) on one of the two marine heatwave treatments (29.5°C or 30.5°C). All treatments were tested at 2- and 4 weeks in treatment and then at 2-weeks post-treatment (all treatments back to control temperature).

Contrast				
A	B	df	t.ratio	p.value
28.5°C-2weeks	29.5°C-2weeks	97	-1.06	0.978
28.5°C-2weeks	30.5°C-2weeks	97	-0.75	0.998
28.5°C-2weeks	28.5°C-4weeks	97	-0.92	0.991
28.5°C-2weeks	29.5°C-4weeks	97	-0.63	0.999
28.5°C-2weeks	30.5°C-4weeks	97	-0.86	0.994
28.5°C-2weeks	28.5°C-2weeks Post	97	0.76	0.998
28.5°C-2weeks	29.5°C-2weeks Post	97	-3.96	**0.004**
28.5°C-2weeks	30.5°C-2weeks Post	97	-3.98	**0.004**
29.5°C-2weeks	30.5°C-2weeks	97	0.31	1.000
29.5°C-2weeks	28.5°C-4weeks	97	0.14	1.000
29.5°C-2weeks	29.5°C-4weeks	97	0.43	1.000
29.5°C-2weeks	30.5°C-4weeks	97	0.20	1.000
29.5°C-2weeks	28.5°C-2weeks Post	97	1.82	0.667
29.5°C-2weeks	29.5°C-2weeks Post	97	-2.90	0.101
29.5°C-2weeks	30.5°C-2weeks Post	97	-2.92	0.097
30.5°C-2weeks	28.5°C-4weeks	97	-0.17	1.000
30.5°C-2weeks	29.5°C-4weeks	97	0.12	1.000
30.5°C-2weeks	30.5°C-4weeks	97	-0.11	1.000
30.5°C-2weeks	28.5°C-2weeks Post	97	1.51	0.847
30.5°C-2weeks	29.5°C-2weeks Post	97	-3.21	**0.045**
30.5°C-2weeks	30.5°C-2weeks Post	97	-3.23	**0.043**
28.5°C-4weeks	29.5°C-4weeks	97	0.29	1.000
28.5°C-4weeks	30.5°C-4weeks	97	0.06	1.000
28.5°C-4weeks	28.5°C-2weeks Post	97	1.68	0.756
28.5°C-4weeks	29.5°C-2weeks Post	97	-3.04	0.071
28.5°C-4weeks	30.5°C-2weeks Post	97	-3.06	0.067
29.5°C-4weeks	30.5°C-4weeks	97	-0.23	1.000
29.5°C-4weeks	28.5°C-2weeks Post	97	1.40	0.897
29.5°C-4weeks	29.5°C-2weeks Post	97	-3.33	**0.032**
29.5°C-4weeks	30.5°C-2weeks Post	97	-3.35	**0.031**
30.5°C-4weeks	28.5°C-2weeks Post	97	1.63	0.788
30.5°C-4weeks	29.5°C-2weeks Post	97	-3.10	0.061
30.5°C-4weeks	30.5°C-2weeks Post	97	-3.12	0.058
28.5°C-2weeks Post	29.5°C-2weeks Post	97	-4.72	**0.000**
28.5°C-2weeks Post	30.5°C-2weeks Post	97	-4.74	**0.000**
29.5°C-2weeks Post	30.5°C-2weeks Post	97	-0.02	1.000

Table S2. Tukey's post-hoc test on the recovery time of *Lutjanus carponotatus*. Adults were held either control (28.5°C) on one of the two marine heatwave treatments (29.5°C or 30.5°C). All treatments were tested at 2- and 4 weeks in treatment and then at 2-weeks post-treatment (all treatments back to control temperature).

Contrast				
A	B	df	t.ratio	p.value
28.5°C-2weeks	29.5°C-2weeks	97	-4.58	**0.000**
28.5°C-2weeks	30.5°C-2weeks	97	-7.32	**0.000**
28.5°C-2weeks	28.5°C-4weeks	97	0.03	1.000
28.5°C-2weeks	29.5°C-4weeks	97	-3.89	**0.006**
28.5°C-2weeks	30.5°C-4weeks	97	-3.73	**0.010**
28.5°C-2weeks	28.5°C-2weeks Post	97	-0.13	1.000
28.5°C-2weeks	29.5°C-2weeks Post	97	0.24	1.000
28.5°C-2weeks	30.5°C-2weeks Post	97	0.62	0.999
29.5°C-2weeks	30.5°C-2weeks	97	-2.73	0.150
29.5°C-2weeks	28.5°C-4weeks	97	4.61	**0.000**
29.5°C-2weeks	29.5°C-4weeks	97	0.70	0.999
29.5°C-2weeks	30.5°C-4weeks	97	0.86	0.995
29.5°C-2weeks	28.5°C-2weeks Post	97	4.45	**0.001**
29.5°C-2weeks	29.5°C-2weeks Post	97	4.82	**0.000**
29.5°C-2weeks	30.5°C-2weeks Post	97	5.20	**0.000**
30.5°C-2weeks	28.5°C-4weeks	97	7.34	**0.000**
30.5°C-2weeks	29.5°C-4weeks	97	3.43	**0.024**
30.5°C-2weeks	30.5°C-4weeks	97	3.59	**0.015**
30.5°C-2weeks	28.5°C-2weeks Post	97	7.18	**0.000**
30.5°C-2weeks	29.5°C-2weeks Post	97	7.56	**0.000**
30.5°C-2weeks	30.5°C-2weeks Post	97	7.93	**0.000**
28.5°C-4weeks	29.5°C-4weeks	97	-3.91	**0.005**
28.5°C-4weeks	30.5°C-4weeks	97	-3.75	**0.009**
28.5°C-4weeks	28.5°C-2weeks Post	97	-0.16	1.000
28.5°C-4weeks	29.5°C-2weeks Post	97	0.21	1.000
28.5°C-4weeks	30.5°C-2weeks Post	97	0.59	1.000
29.5°C-4weeks	30.5°C-4weeks	97	0.16	1.000
29.5°C-4weeks	28.5°C-2weeks Post	97	3.75	**0.009**
29.5°C-4weeks	29.5°C-2weeks Post	97	4.13	**0.002**
29.5°C-4weeks	30.5°C-2weeks Post	97	4.50	**0.001**
30.5°C-4weeks	28.5°C-2weeks Post	97	3.59	**0.015**
30.5°C-4weeks	29.5°C-2weeks Post	97	3.97	**0.004**
30.5°C-4weeks	30.5°C-2weeks Post	97	4.34	**0.001**
28.5°C-2weeks Post	29.5°C-2weeks Post	97	0.38	1.000
28.5°C-2weeks Post	30.5°C-2weeks Post	97	0.75	0.998
29.5°C-2weeks Post	30.5°C-2weeks Post	97	0.38	1.000

Table. S3. Tukey's post-hoc test on the EPOC of *Lutjanus carponotatus*. Adults were held either control (28.5°C) on one of the two marine heatwave treatments (29.5°C or 30.5°C). All treatments were tested at 2- and 4 weeks in treatment and then at 2-weeks post-treatment (all treatments back to control temperature).

Contrast				
A	B	df	t.ratio	p.value
28.5°C-2weeks	29.5°C-2weeks	97	-4.28	**0.001**
28.5°C-2weeks	30.5°C-2weeks	97	-5.90	**0.000**
28.5°C-2weeks	28.5°C-4weeks	97	-0.71	0.999
28.5°C-2weeks	29.5°C-4weeks	97	-7.00	**0.000**
28.5°C-2weeks	30.5°C-4weeks	97	-6.33	**0.000**
28.5°C-2weeks	28.5°C-2weeks Post	97	-0.38	1.000
28.5°C-2weeks	29.5°C-2weeks Post	97	-0.82	0.996
28.5°C-2weeks	30.5°C-2weeks Post	97	-2.03	0.525
29.5°C-2weeks	30.5°C-2weeks	97	-1.61	0.794
29.5°C-2weeks	28.5°C-4weeks	97	3.57	**0.015**
29.5°C-2weeks	29.5°C-4weeks	97	-2.72	0.156
29.5°C-2weeks	30.5°C-4weeks	97	-2.05	0.516
29.5°C-2weeks	28.5°C-2weeks Post	97	3.90	**0.005**
29.5°C-2weeks	29.5°C-2weeks Post	97	3.46	**0.022**
29.5°C-2weeks	30.5°C-2weeks Post	97	2.25	0.384
30.5°C-2weeks	28.5°C-4weeks	97	5.19	**0.000**
30.5°C-2weeks	29.5°C-4weeks	97	-1.10	0.973
30.5°C-2weeks	30.5°C-4weeks	97	-0.43	1.000
30.5°C-2weeks	28.5°C-2weeks Post	97	5.52	**0.000**
30.5°C-2weeks	29.5°C-2weeks Post	97	5.08	**0.000**
30.5°C-2weeks	30.5°C-2weeks Post	97	3.86	**0.006**
28.5°C-4weeks	29.5°C-4weeks	97	-6.29	**0.000**
28.5°C-4weeks	30.5°C-4weeks	97	-5.62	**0.000**
28.5°C-4weeks	28.5°C-2weeks Post	97	0.33	1.000
28.5°C-4weeks	29.5°C-2weeks Post	97	-0.11	1.000
28.5°C-4weeks	30.5°C-2weeks Post	97	-1.32	0.921
29.5°C-4weeks	30.5°C-4weeks	97	0.67	0.999
29.5°C-4weeks	28.5°C-2weeks Post	97	6.62	**0.000**
29.5°C-4weeks	29.5°C-2weeks Post	97	6.18	**0.000**
29.5°C-4weeks	30.5°C-2weeks Post	97	4.96	**0.000**
30.5°C-4weeks	28.5°C-2weeks Post	97	5.95	**0.000**
30.5°C-4weeks	29.5°C-2weeks Post	97	5.51	**0.000**
30.5°C-4weeks	30.5°C-2weeks Post	97	4.29	**0.001**
28.5°C-2weeks Post	29.5°C-2weeks Post	97	-0.44	1.000
28.5°C-2weeks Post	30.5°C-2weeks Post	97	-1.66	0.772
29.5°C-2weeks Post	30.5°C-2weeks Post	97	-1.22	0.951

Table. S4. Tukey's post-hoc test on the recovery rate of *Lutjanus carponotatus*. Adults were held either control (28.5°C) on one of the two marine heatwave treatments (29.5°C or 30.5°C). All treatments were tested at 2- and 4 weeks in treatment and then at 2-weeks post-treatment (all treatments back to control temperature).

Contrast				
A	B	df	t.ratio	p.value
28.5°C-2weeks	29.5°C-2weeks	97	1.42	0.887
28.5°C-2weeks	30.5°C-2weeks	97	2.17	0.435
28.5°C-2weeks	28.5°C-4weeks	97	-1.11	0.971
28.5°C-2weeks	29.5°C-4weeks	97	-1.32	0.924
28.5°C-2weeks	30.5°C-4weeks	97	-1.05	0.980
28.5°C-2weeks	28.5°C-2weeks Post	97	-0.33	1.000
28.5°C-2weeks	29.5°C-2weeks Post	97	-1.63	0.785
28.5°C-2weeks	30.5°C-2weeks Post	97	-4.61	**0.000**
29.5°C-2weeks	30.5°C-2weeks	97	0.75	0.998
29.5°C-2weeks	28.5°C-4weeks	97	-2.53	0.230
29.5°C-2weeks	29.5°C-4weeks	97	-2.74	0.149
29.5°C-2weeks	30.5°C-4weeks	97	-2.47	0.260
29.5°C-2weeks	28.5°C-2weeks Post	97	-1.75	0.713
29.5°C-2weeks	29.5°C-2weeks Post	97	-3.05	0.069
29.5°C-2weeks	30.5°C-2weeks Post	97	-6.03	**0.000**
30.5°C-2weeks	28.5°C-4weeks	97	-3.28	**0.037**
30.5°C-2weeks	29.5°C-4weeks	97	-3.48	**0.020**
30.5°C-2weeks	30.5°C-4weeks	97	-3.22	**0.044**
30.5°C-2weeks	28.5°C-2weeks Post	97	-2.50	0.245
30.5°C-2weeks	29.5°C-2weeks Post	97	-3.80	**0.007**
30.5°C-2weeks	30.5°C-2weeks Post	97	-6.78	**0.000**
28.5°C-4weeks	29.5°C-4weeks	97	-0.21	1.000
28.5°C-4weeks	30.5°C-4weeks	97	0.06	1.000
28.5°C-4weeks	28.5°C-2weeks Post	97	0.78	0.997
28.5°C-4weeks	29.5°C-2weeks Post	97	-0.52	1.000
28.5°C-4weeks	30.5°C-2weeks Post	97	-3.50	**0.019**
29.5°C-4weeks	30.5°C-4weeks	97	0.27	1.000
29.5°C-4weeks	28.5°C-2weeks Post	97	0.98	0.986
29.5°C-4weeks	29.5°C-2weeks Post	97	-0.32	1.000
29.5°C-4weeks	30.5°C-2weeks Post	97	-3.29	**0.036**
30.5°C-4weeks	28.5°C-2weeks Post	97	0.72	0.998
30.5°C-4weeks	29.5°C-2weeks Post	97	-0.58	1.000
30.5°C-4weeks	30.5°C-2weeks Post	97	-3.56	**0.016**
28.5°C-2weeks Post	29.5°C-2weeks Post	97	-1.30	0.929
28.5°C-2weeks Post	30.5°C-2weeks Post	97	-4.28	**0.001**
29.5°C-2weeks Post	30.5°C-2weeks Post	97	-2.98	0.084
